# Implementing organ-on-chip in a next-generation risk assessment of chemicals: a review

**DOI:** 10.1007/s00204-022-03234-0

**Published:** 2022-02-01

**Authors:** Katharina S. Nitsche, Iris Müller, Sophie Malcomber, Paul L. Carmichael, Hans Bouwmeester

**Affiliations:** 1 Division of Toxicology, Wageningen University, P.O. Box 8000, 6700 EA Wageningen, The Netherlands; 2 Unilever Safety and Environmental Assurance Centre, Colworth Science Park, Sharnbrook, Bedfordshire MK44 1LQ UK

**Keywords:** Microfluidics, Organ-on-chip, Next-generation risk assessment, Skin-on-chip, Gut-on-chip, Liver-on-chip

## Abstract

Organ-on-chip (OoC) technology is full of engineering and biological challenges, but it has the potential to revolutionize the Next-Generation Risk Assessment of novel ingredients for consumer products and chemicals. A successful incorporation of OoC technology into the Next-Generation Risk Assessment toolbox depends on the robustness of the microfluidic devices and the organ tissue models used. Recent advances in standardized device manufacturing, organ tissue cultivation and growth protocols offer the ability to bridge the gaps towards the implementation of organ-on-chip technology. Next-Generation Risk Assessment is an exposure-led and hypothesis-driven tiered approach to risk assessment using detailed human exposure information and the application of appropriate new (non-animal) toxicological testing approaches. Organ-on-chip presents a promising in vitro approach by combining human cell culturing with dynamic microfluidics to improve physiological emulation. Here, we critically review commercial organ-on-chip devices, as well as recent tissue culture model studies of the skin, intestinal barrier and liver as the main metabolic organ to be used on-chip for Next-Generation Risk Assessment. Finally, microfluidically linked tissue combinations such as skin–liver and intestine–liver in organ-on-chip devices are reviewed as they form a relevant aspect for advancing toxicokinetic and toxicodynamic studies. We point to recent achievements and challenges to overcome, to advance non-animal, human-relevant safety studies.

## Introduction

Organ-on-chip (OoC) technologies attract increasing interest as human, physiologically relevant in vitro testing systems to be incorporated in a Next-Generation Risk Assessment (NGRA) of chemicals. OoC are small scale devices designed for dynamic human cell culture that can simulate different microenvironments and functions in such a way that the cells can behave as naturally as possible (i.e., more in vivo-like) (Mummery et al. 2020). The “natural” microenvironment and functions are introduced to the cells in the OoCs, using microfluidic flow, 3D tissue reconstruction and the use of multiple cell types and cell sources. OoC hardware devices vary on the materials used (e.g., rubber, plastic, silicone, glass), layout (open or closed culture compartments), perfusion (active or passive) and can provide different support for cell culturing on chip (e.g., stretch, peristaltic, contraction dynamics etc.). In the last decade, numerous published microfluidic chip approaches have accelerated the innovation and commercial large-scale production of these devices (see “Commercially manufactured OoC devices”). This resulted in an increasing infrastructural development for biomedical laboratories without the need of in-house microfluidic designing expertise. In parallel, recent publications using human cells in OoC devices underpin the advances in biology by demonstrating that the induced biochemical and mechanical cues improve functional and structural characteristics of tissue cultures. The combination of both, tissue function with flow dynamics in 3D architecture, may significantly contribute to the transition of animal-free approaches for regulatory safety assessment (e.g., development of adverse outcome pathways) (Heringa et al. 2020). In addition, OoC might also accelerate new approach methodology acceptance for NGRA, defined as human-relevant, exposure-led, hypothesis driven risk assessment approach that integrates in silico, in chemico and in vitro approaches for assessing effects on human health (Berggren et al. 2017; Dent et al. 2018; Thomas et al. 2019). In this tiered approach, OoC systems have value in the higher tier ab initio approach for targeted testing, biokinetic refinements, as well as the estimation of the points of departure, uncertainty, margin of safety and extrapolation (Berggren et al. 2017). Yet, there is a clear consensus in the OoC community, (consumer) industry and regulatory bodies on the need for standardisation to advance the field (Piergiovanni et al. 2021).

The review we focus on data available from OoC manufacturer websites, as well as on the search for current (2016–2021) tissue-specific studies including different cell lines and types in skin, intestine and gut models. With the review, we aim to evaluate the features and robustness of the currently available manufactured OoC devices and skin, intestine and liver tissue models that can be used as part of the NGRA toolbox and at higher tier testing on-chip (see selection in tables and figures). First, we describe commercially manufactured OoC devices that thus have a (more) standardized design and critically discuss the applicability of these devices for toxicological studies. Next, some promising achievements with microfluidic in vitro tissue culturing approaches are highlighted. For this analysis, we focussed on two important biological barriers, the skin and gastrointestinal epithelium, as these are of particular relevance for safety assessment that cannot use laboratory animal-derived data. In addition, liver models were reviewed as they represent the most metabolically active tissue, which is a key characteristic if systemic toxicology is considered. Finally, fluidically linked tissue combinations such as skin–liver and intestine–liver in OoC devices are reviewed as they form an innovative aspect for advancing and integrating kinetics studies, which are needed to increase the physiological relevance of in vitro models. We conclude this review by listing additional research and standardization that are required to qualify OoC as fit-for-purpose systems in a NGRA toolbox.

## Commercially manufactured OoC devices

**Fig. 1:**
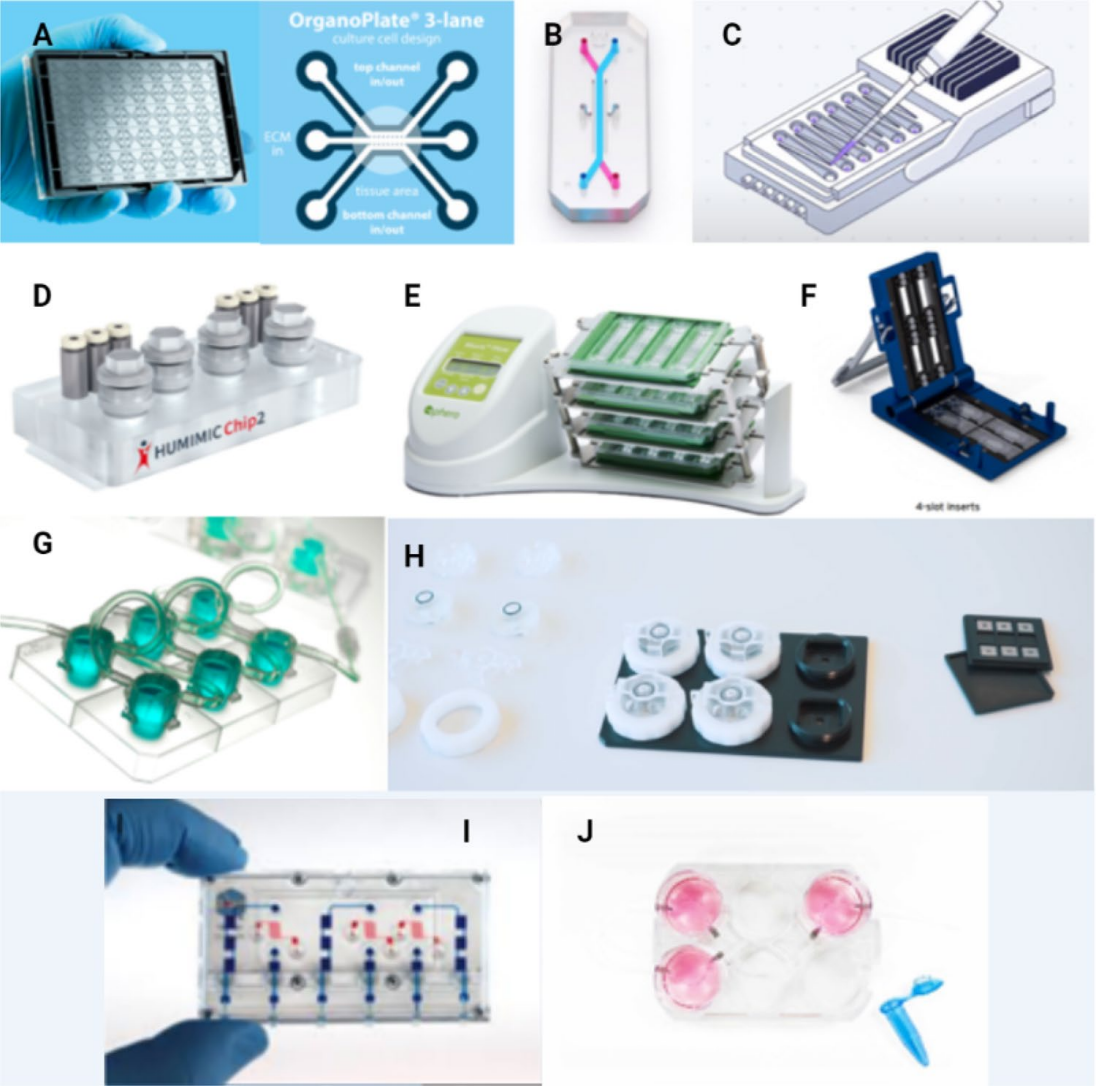
Examples of commercially available OoC devices for different research applications **A** OrganoPlate® 3-lane|Mimetas (2020) **B** Organs-on-Chips Technology|Emulate (2020) **C** PhysioMimix™|CN BIO Innovations (2020) **D** HUMIMIC Chip2|TissUse GmbH (2022) **E** Akura™ Flow: Transforming Drug Discovery and Development with Body-on-a-Chip Technology|InSphero (2020) **F** Organ-on-a-chip|Micronit (2020) **G** The QV900|Ideal for high-content experiments and industrial use|Kirkstall Ltd (2020) **H** Products-Bi/ond (2020) **I** The ParVivo™ Organ-on-Chip Technology|Nortis Bio (2020) **J** HuDMOP®|IONTOX (2022). All pictures taken from the websites of manufacturers (see references)

Due to the broad need for human-relevant in vitro approaches for different research applications, the development of novel devices is constantly stimulated. A decade after the first successfully lab-fabricated OoC, a wide variety of commercially manufactured hardware became available to emulate a more natural microenvironment for in vitro studies (Mummery et al. 2020). The hardware design of an OoC is dictated by the microenvironment required for optimum cellular functions, the monitoring parameters and the research application. The design of the hardware in turn determines the material selection (e.g., rubber, plastic, silicone and glass) with its associated fabrication technique (e.g., Lithography, 3D printing) and the options for stimulating and sensing (e.g., mechanical, optical), as well as the interfacing layout (e.g., open- or closed culture compartment accessibility, liquid perfusion) (Kurth et al. 2020). Based on the material selection and interfacing, we will discuss commercially manufactured devices, as shown in Fig. 1.

## Materials used for OoC devices

Not all materials are suitable for an OoC fabrication as they must support the growth of functional cells and allow the study of the biological model in this simplified microenvironment (Kurth et al. 2020). The material selection must consider the creation of appropriate culture chambers and with fluidic connection, induced forces and stiffness to recreate biological functions, as well as potentially adding electrical stimuli and actuation to control and observe the created biological tissue model. The culture chambers are the advanced compartments that are often separated by porous membranes to host the cultured tissue, partially with the help of a biological scaffold. In these culture compartments the cell tissue is provided with the necessary nutrients, waste products are removed and a (bio)chemical environments (e.g., gradients) can be simulated (e.g., oxygen, carbon dioxide, acidity etc.) (Mummery et al. 2020). It is rare that a single material can be used to fabricate an entire complex OoC device, including the culture compartment and fluidic connections with mechanics and sensors, as multiple criteria must be considered in the material selection and integration. Material criteria include the biocompatibility, sterilization, physiochemical properties, material function on the device and eventually the costs. For (novel) material used in OoC, biocompatibility implies that the material supports appropriate cellular activity without undesired or harmful biological effects (Zhang et al. 2018; Kurth et al. 2020). As the material is in direct or close contact with cells, it is necessary to avoid contamination, and therefore, it is important that the material must withstand sterilisation techniques. Essential physiochemical properties are optical transparency for observation, gas permeability for cells requiring oxygen, lowest possible absorption of molecules, chemical and thermal resistance, as well as stiffness. As mentioned earlier in this section, these requirements largely depend on the chip design and research application. Currently, common materials used for creating OoC devices are silicone substrates and polymers (e.g., PDMS), resins and glass partly meet the required criteria [see Azizipour et al. (2020) and Ding et al. (2020)]. Further improvements are needed and novel (hybrid) material may fulfil the various engineering and biological requirements (Ding et al. 2020; Grant et al. 2021).

## Open and closed tissue culture compartments

All device manufacturers strive for different layouts to target different research applications. This leads to at least two major design differences in layout for the tissue culture compartments access: an open or closed access. As to be seen in the devices C, D, E, F, G, H, J, as depicted in Fig. 1, the open culture compartment offers a direct access for seeding, sampling, dosing and analysis. This layout partially facilitates air–liquid interfacing for skin and gut, the seeding of bigger cell aggregates such as liver spheroids and the layering of dermal and intestinal cell sheets on an insertable membrane as reviewed by Berthier et al. (2019). The closed layouts (see Fig. 1A, B, I) can mimic better a closed 3D organ architectures and mechanical forces, such as flow and stretch for intestinal, liver and vascular tissues. However, this closed layout complicates the extraction of cell samples for analysis (Bhatia and Ingber 2014). The open or closed tissue culture compartment access also impacts the requirement for tubing and pumps for fluidic perfusion.

## Fluidic perfusion of the devices

Fluidic perfusion ensures the continuous supply of nutrients and removal of waste products from the cell culture. In addition, perfusion delivers mechanical stimuli by generating laminar, pulsative and interstitial shear stress along the microfluidic channel thus recreating living cell environments with biochemical gradients and cell signaling (Rothbauer et al. 2018). Perfusion methods are either passive or active through direct integration or plugged-in system to actuate the fluidic flow as reviewed by Kurth et al. (2020). The simplest and least-expensive method is gravity-driven which uses mostly a rocking platform to induce the passive flow (see Fig. 1A, E). The induced bidirectional flow results from the difference in liquid height between the fluid inlet and outlet within an closed culture compartments (Kaarj and Yoon 2019). Mechanical active perfusion through directly integrated or plugged-in pneumatic and peristaltic pumps offer a simple solution to deliver culture medium from source to waste or to recirculate the culture medium. A number of cell tissues can be perfused in parallel, depending either on the amount of tubing which can be coiled around the peristaltic pump or the microvalve amount connected to the pressure controlled pneumatic pump (also on-chip) (see Fig. 1B–D, F–J). Both, passive and active perfusion inherent limitations in their fluid handling as reviewed by Soenksen et al. (2018). For example, mechanical pumps (also directly on-chip) connected to open wells or channels usually do not deliver robust steady-state flows for long periods of time as they rely on pulsative flows (e.g., directly integrated or plug-in pumps), extra tubing and are more susceptible to contamination and air-bubbles (Mäki et al. 2015; Soenksen et al. 2018). Furthermore, devices relying on active perfusion are equipped with extra instruments (e.g., pressure controls, sensors) and use either connective tubing or stiff monolithic design material that might impact the cell culture. However, media flow actuation with tubing or a monolithic design are so far the only approaches to interconnect individual culture chips. The tunable flow enables the control and circulation of media with secreted molecules by perfusing the entire system with common medium which paves the way to engineer complex human physiology on chip (Renggli and Frey 2020). Passive perfusion through gravity does not use extra tubing and instruments but is transient in nature and prone to performance variation (e.g., fabrication error, use-induced stress, trapped air bubbles). In addition, closed passive perfused systems can affect the chemical distribution rates as the combination of high plastic exposure with lower fluidic exchange and a lack of headspace may accumulate chemicals, especially after repeated exposure (Kramer et al. 2015; Proença et al. 2021). Future experiments for both, active and passive perfused systems, should address the potentially affected chemical biokinetics to provide clarity on diffusion rates.

## Engineering human tissue functionality on chip

In the last years, the work is progressing on new approach methodologies for human relevant biokinetic predictions that move away from animal experimentation towards in silico and novel cell culture technologies (Punt et al. 2020). The use of animal data in human risk assessment raises concerns as animal tissue physiology does not always recapitulate human tissue physiology. In contrast, some static in vitro models with human cells may not represent the sensitive cellular microenvironment required for physiologically relevant simulations. OoC technology in combination with advance human cell models potentially offers a promising alternative to improve in vitro experiments by introducing biological functions, such as microfluidic shear stress and a 3D microenvironment. Despite the great promises of OoC, examples of successful application of OoC for NGRA are scarce due to cost, throughput, general OoC availability and cell culture challenges (Rusyn and Roth 2021; Low et al. 2021).

Next, we will discuss the engineering of a controlled 3D environment and three key human organ tissue systems in the culture compartments. We will elaborate on skin, intestine and liver models that are equivalent to at least the smallest functional unit of each organ (Ronaldson-Bouchard and Vunjak-Novakovic 2018; Jensen and Teng 2020). There are two critical factors which need to be addressed while engineering a tissue with organ-specific function on chip for a NGRA: (1) the establishment of a 3D architecture in the culture compartments and (2) the choice of cell line. In this section we discuss the significance of the above two factors and how they affect the functional capacity of the recreated tissue.

Single cells type monolayers might underrepresent the functional complexity as exhibited in the in vivo environment; however, a shift can be observed to improve the culture environment through the implementation of new approach methodologies (Punt et al. 2020). One new in vitro approach includes 3D cell culturing to recreate an anatomical architecture of a tissue of interest. Several studies have shown that upon recreating a 3D architecture, the cultured cells have improved characteristics in morphology, viability, differentiation, metabolic capacity as well as transporter and gene expression levels (Duval et al. 2017; Curto et al. 2017; Theobald et al. 2018; Lembong et al. 2018; Lee and Jun 2019; Jensen and Teng 2020). Two directions can be observed in recreating 3D architectures in OoCs. First, scaffold free techniques such as hanging drops, magnetic levitation and spheroid microplates with ultra-low attachment coating enable the cells to freely grow prior to seeding in OoCs. This technique is especially applicable for open accessible culture compartments as this layout allows direct seeding of bigger aggregates as demonstrated for liver spheroids (Lasli et al. 2019; Jang et al. 2019a; Kostrzewski et al. 2020; Tao et al. 2021). Second, scaffold-based techniques which use hard material-based polymers or hydrogel supports that mimic the extra cellular matrix (ECM) and enables the cells to properly attach and differentiate (Jensen and Teng 2020). The ECM biomaterial can be tumour cell-derived (e.g., collagen, Matrigel), purified protein, polysaccharide (e.g., collagens, alginate, bacterial cellulose) or produced synthetically (e.g., polyethylene glycol). Notably, all biomaterial will impact the intracellular signalling as well as the chemical distribution in the cell system (Gjorevski and Lutolf 2017; Hinman et al. 2020). Especially OoC have very particular chemical distribution processes that need to be addressed for better translatability, as reviewed by Proença et al. (2021). The review concludes that chemical distribution simulations are important for the validation, as part of chemical hazard identification (Proença et al. 2021). Furthermore, scaffold-free and scaffolded techniques exploit the self-assembling capacity of cells. Different organ systems require specific 3D scaffolds, and cell types to allow for targeted functional tissue applications on chip and, therefore, require unique microfabrication techniques (Duval et al. 2017). Recent advanced in microfabrication techniques such as 3D printing offer a higher potential to recreate a controlled and reproducible 3D architecture (Zhao et al. 2019).

## Creating the best biology on chip

Selecting the cell source is a critical aspect to consider for the engineering of a functional, scalable and reproducible organ tissue equivalent (Renggli and Frey 2020). The type of human cells to use largely depends on the emulation of the desired physiological function along with the cell type availability, cultivability, time line of the study, budget and availability of established protocols. Immortalized cell lines, primary cell cultures and derivatives of adult or induced pluripotent stem cells are classes of cells that can be utilized for tissue recreation. Advantages and limitations vary depending on the target organ and importantly on the research question.

Cell lines are widely used in toxicological proof-of-concept studies, because they are robust, easy to culture, well-characterized, affordable and highly proliferative. However, cancerous cell lines often exhibit significant genotypical and phenotypical abnormalities such as lack of metabolic capacity in terms of CYP450 gene expression and other metabolic enzymes, as well as lack of expression of protein transporters, potentially limiting their ability to reproduce physiological cell behaviour (Gillet et al. 2013).

In contrast to cell line-based models, primary cell cultures obtained from fresh tissue are considered a gold standard for in vitro models, because they resemble the in vivo cells the most. Primary cultures of certain cell types consist of a heterogenous cell population at various stages of differentiation and maturation (Verma et al. 2020). Once the cells are terminally differentiated, they may attach and remain viable in culture but will not proliferate anymore and nearly instantly dedifferentiate. The limited proliferative capacity is termed as replicative senescence, causing the major disadvantage of using primary cells in OoC (Cristofalo et al. 2004). Primary cell lines inherit the donor genotype that enables investigation of specific features of vulnerable groups but is a limitation for the generic assessment of molecular pathways and metabolism broader populations (Castell et al. 2006; Ertel et al. 2006).

The use of stem cell-derived cell culture models is gaining pace in toxicological research and continues to advance together with microfluidic culturing. Most stem cell-derived models in OoC are based on induced pluripotent stem cells (iPSC) as the microfluidic devices can direct differentiation (Yaqing Wang et al. 2018a, b; Ramme et al. 2019; Naumovska et al. 2020). The major iPSC-stem cell advantage is the usual normal karyotype and their derivation from human material, such as from biopsies, blood draws and urine. Upon reprogramming the derived human material, the stem cells may be selectively differentiated into multiple tissue specific-cell lineages, creating a replenishable source of cells (Wnorowski et al. 2019). Same as primary cells, iPSCs inhere the donor genotype which contributes to experimental variability and affect reproducibility of experiments. The genotypic and phenotypic differences make them on one hand ideal to study chemical responses for susceptible groups, whereas it might complicate mode of action studies for broader populations. Nevertheless, stem cells are in demand for NGRA studies, leading to a rapid development of culture protocols to overcome the largely fetal-like phenotype (Bulutoglu et al. 2020). The major challenge is the establishment of a robust and reproducible approach to maintain, differentiate and mature iPSC cell lines in vitro*.* Importantly, recent work by the groups of Bulutoglu and Sakolish raise confidence in lab-to-lab comparable and primary cell-like performing iPSC-derived hepatocytes in OoC (Bulutoglu et al. 2020; Sakolish et al. 2021). Notably, also direct on-chip culturing techniques were performed using iPSC-derived intestinal organoids. The derived cells exhibited organ-specific function in a quicker and resource-efficient manner (Naumovska et al. 2020). Despite current obstacles, stem cell culturing is expected to synergistically advance with OoC technology towards more robust human physiological models (Low et al. 2021).

## Advancing skin, intestine and liver tissue cultures on chip for next-generation risk assessment

In this section we highlight promising advances for in vitro tissue culturing approaches that resulted in show case models. To do so, we focus on two important biological barriers, the skin and the gastrointestinal epithelium as important barriers for chemicals. In addition, we include liver-on-a-chip models as liver is the main metabolic active tissue and, therefore, highly relevant to include in NGRA. Finally, fluidically linked tissue combinations on-chip such as skin–liver and intestine–liver are reviewed as they form an innovative aspect for advancing and simulating kinetics for in silico modelling.

## Application of skin-on-a-chip in next-generation risk assessment of chemicals

The skin is the largest organ of the human body and it is in direct contact with the outside environment. Thus, a healthy skin features barrier characteristics and thereby regulates the body temperature, retains moisture and protects against microbes and chemicals (Gauglitz and Schauber 2014). The human skin consists of three tissue layers—epidermis, dermis and subcutaneous layer. The stratum corneum is the epidermal top layer and is composed of dead skin cells and functions as the primary barrier. The epidermis is a dense and poorly vascularized region that mainly consists of keratinocytes (KC) with few pigment-producing dendritic cells (DC). Major immune cells in the epidermis are Langerhans cells (LCs) and dendritic epidermal T-cells (DETC). Below the epidermis lies the dermis layer which consists of a highly vascularized fibrotic layer which is low in cell density but rich in collagen and elastin fibres. Fibroblasts are the major cell type alongside with scattered immune cells. Finally, the deepest layer is the subcutaneous layer of fat that supplies nutrients to the outer layers. The epidermis and dermis play a major role in absorption, distribution, metabolism of xenobiotics as well as generate an immune response against xenobiotics. Therefore, these two layers are in focus for recreating better human relevant skin–tissue models (Chong et al. 2013). Within the NGRA toolbox for skin-contact materials, better skin models find value in the risk assessment of traditional endpoints, such as irritation, corrosion, phototoxicity, skin sensitization, as well as understanding and improving exposure estimations, stress pathways and metabolism (Gilmour et al. 2020; Baltazar et al. 2020).

**Fig. 2:**
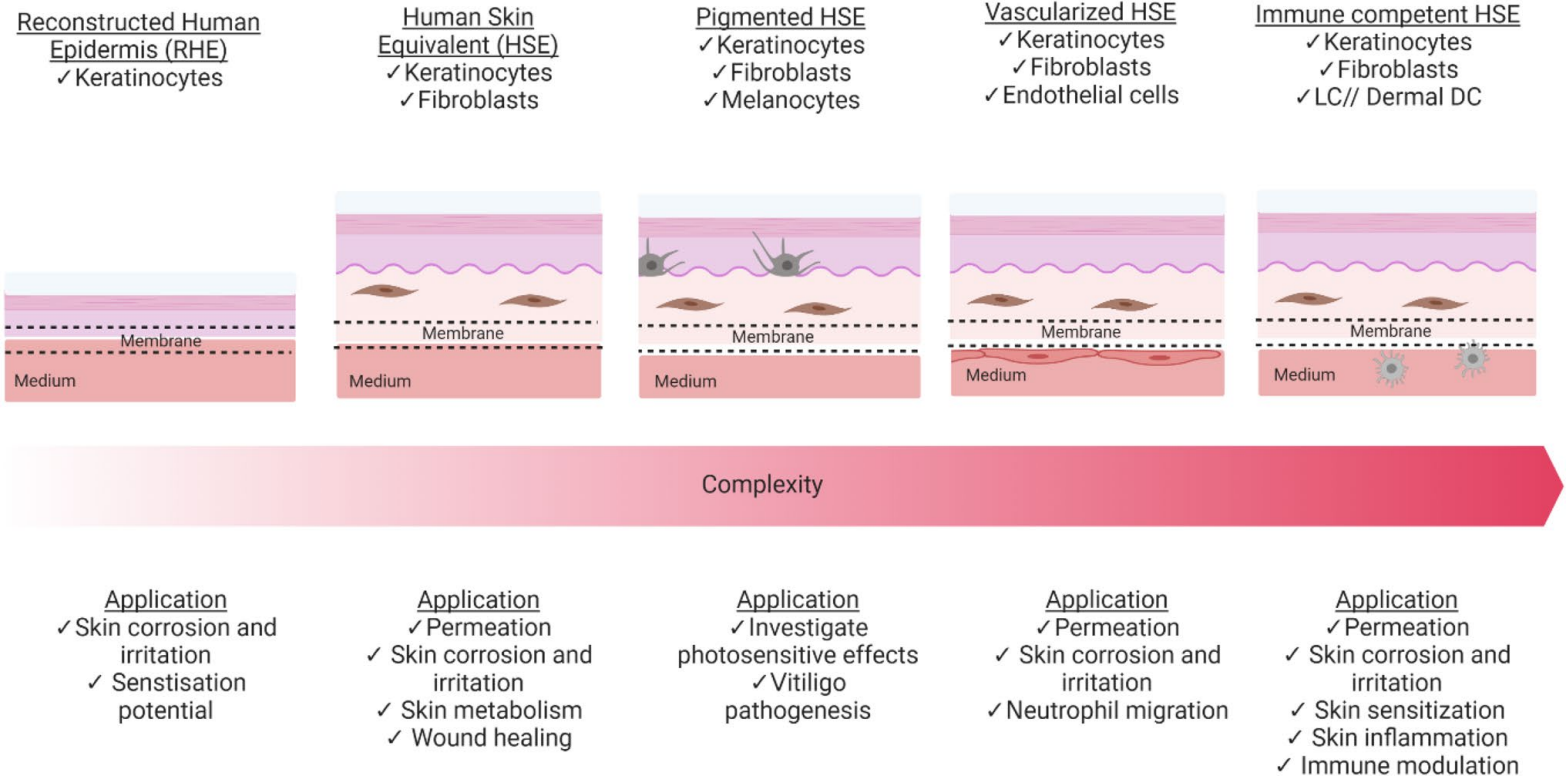
Summary of selected 3D in vitro skin tissue models, depicted with increasing biological complexity and their research applicability and predictability for NGRA using an open access OoC device for air–liquid culturing

Current skin research relies on the use of ex vivo mimetic models as gold standard but their use is not always possible due to ethical concerns, regulatory issues and variability, because samples are usually obtained from different anatomical sites (Moniz et al. 2020). Hence, engineered human skin tissues have been widely adopted for assessment of local toxicity in the skin. Notably, this resulted in harmonised in vitro testing by the newly adopted OECD testing guidelines which now involve human-based in vitro skin tissue models for chemical evaluation (Ng and Yeong 2019; OECD 2021a, b, c). To evaluate the safety of chemicals, an in vitro 3D skin tissue can be either readily purchased or created by layering cell sheets. Then, these skin tissue models can be cultured dynamically in an open-top OoC device to be consequently lifted for creating an air–liquid interface (ALI) on the cell layer, as this forms the stratum corneum layer of the epidermis. In the past decade, various skin tissue models with different levels of biological complexity have been developed with immortalized cell lines, primary cells and stem cells (see Fig. 2) (Kandarova and Hayden 2021).

The simplest in vitro skin model is the Reconstructed Human Epidermis (RHE, see Fig. 2; Table 1) consisting only of keratinocytes cultured on a collagen matrix at the air–liquid interface. Commercially manufactured RHE models, Episkin^®^, EpiDerm™, epiCS^®^ and SkinEthic™, were first assessed as a predictive model for skin corrosion, but soon they were approved for determination of irritation potential marked through IL-1α and IL-18 release (Gibbs et al. 2013; Ng and Yeong 2019). These RHE models are created with primary human keratinocytes (foreskin or mammary skin tissue) but other RHE models have also been established with immortalized human keratinocytes (HaCat) and iPSC-derived skin cells (Petrova et al. 2016). All of them form a stratified epithelium after around 14 days of culturing under optimal conditions at the ALI (Mathes et al. 2014). RHE models are especially acknowledged for their reproducibility but they do not meet the requirement for permeation studies. Schäfer-Korting et al. (2006) showed that permeation of caffeine and testosterone using RHE is overestimated compared to the human epidermis. Therefore, RHE has limitedly utility for NGRA studies that require barrier function but may represent a useful tool for corrosion, irritation and skin sensitization potentials (Zhang et al. 2017a; Song et al. 2018; Teimouri et al. 2019; Mehling et al. 2019).

**Table 1:** Selection of skin models that are culturable in microfluidic devices

Type of skin model	Cell line used (in OoC)	Advantage	Potential limitation	Example application from the literature
Reconstructed human epidermis (RHE)	Primary cells	OECD validated for skin irritation and corrosion Highly reproducible Preferred for Comet Assay (Genotoxicity test) Comparable to in vivo keratinocytes	Lower barrier property compared to in vivo and bilayered model Low proliferative capacity (senescence) Single cell type representation Low metabolic capacity Only performed static	Measured release of IL-18 after exposure to a list of 40 skin sensitizers and allergens over 24 h in static environment (Gibbs et al. 2013) Measured Benzo[a]pyrene metabolism and genotoxicity over 48 h in static environment (Brinkmann et al. 2013)
	Immortalized cell line, i.e., HaCaT, hTERT immortalized primary cells	ease of handling, cost and accuracy for chemicals with adequate solubility Widely used for cytotoxicity testing Acceptance criteria for OECD guideline available	Low uptake of chemicals with extremely low solubility Lower barrier property compared to in vivo and bilayered model Single cell type representation	Measured ${Particulate Matter}_{2.5}$-induced cytotoxicity for 24 h (Zhang et al., 2017a, b)
	iPSC-derived monoculture	High proliferative capacity Ideal for disease modelling	possible inter donor variations still requires assessment for evaluation/validation for comparison to adult cells Suspected lower barrier property compared to in vivo and HSE (but still higher than other sources for RHE) Single cell type representation Only performed static	Measured differences between two tested iPSC clones for 3D HSE generation in comparison to primary healthy epidermal model. No exposure. Static environment (Petrova et al. 2016)
Human skin equivalent (HSE)	Primary cells/tissues	Higher barrier property than RHE model (thus more suitable for permeation studies) Addition of fibroblasts essential for wound healing studies Culture of 3 weeks was demonstrated Approved by OECD for several endpoints Show good metabolic capacity Acceptance criteria for OECD guideline available	Still lower barrier property than in vivo	Measured toxic effects of doxorubicin in pumpless microfluid platform for 3 weeks (Abaci et al. 2015) Validated 3D Skin comet assay with Mitomycin C; Cadmium chloride; *N*-ethyl-*N*-nitrourea; 7,12-dimethylbenz(a)thracene; Propyl gallate; Eugenol; Di-(2-ethylhexyl)phthalate; Cyclohexanone over 48 h in static environment (Reisinger et al. 2018) Measured metabolite formation and genotoxicity after (chronic) 2-acetylaminofluorene exposure for 48 h in static environment (Downs et al. 2021)
	Immortalized, i.e., HaCaT, hTERT immortalized primary cells and cocultures	higher barrier property than RHE model (thus more suitable for permeation studies) Addition of fibroblasts essential for wound healing studies Culture of 2 weeks was demonstrated	Still lower barrier property than in vivo HaCaT show lower metabolism than primary cells	Measured caffeine, salicyclic acid and testosterone skin permeation in model containing N/TERT–keratinocytes with primary fibroblasts in microfluid permeation array for 2 weeks (Alberti et al. 2017)
	iPSC-derived	Can be potentially be differentiated into all cell types in the skin High proliferative capacity	Stratum corneum-like structure was not readily observed (= leaky barrier) Time and cost intensive Metabolic capacity not assessed Only performed static	Measured the skin permeation of 5(6)-carboxyfluorescein and fluorescein isothiocyanate dextran 4000 over 360 min in model containing iPSC-derived keratinocytes and fibroblasts in static environment (Naito et al. 2019)
Pigmented reconstructed human skin	Primary cells and co-cultures	Retain many morphological and signalling properties of In situ skin Model for phototoxicity, sun-related effects and vitiligo pathogenesis	Lower growth rate than primary keratinocytes (can lead to either hypopigmentation or scattered pigment patches) Inter individual variations highly likely Only performed static Does not meet acceptance criteria for OECD guideline	Measured impact of UV radiation on dermal and epidermal DNA damage in static environment (Goyer et al. 2019)
	Immortalized, i.e., HaCaT, hTERT immortalized primary cells	Individually well studied cell types Model for phototoxicity, sun-related effects and vitiligo pathogenesis	Limited comparative studies with other pigmented immortalized cell line skin models available Only performed static	Measured whitening efficacy of ginsenoside F1 in HaCaT–MNT-1 coculture for 72 h in static environment (Lee et al. 2019a)
	iPSC	might represent a valuable model system for pigmentary disorders High proliferative capacity	Relatively new system with limited available data Time and cost intensive Only performed static	Measured melanin transfer in all iPSC-derived 3D pigmented HSE in static environment (Gledhill et al. 2015)
Immune competent skin model	Primary cells and co-cultures	Model for permeation, corrosion and irritation, sensitization and inflammation Demonstrate in vivo-like inflammation response Presence might enable earlier detection of sensitizers (and reduce false positives)	Low proliferative capacity and short culturing of peripheral blood mononuclear cells (requires fresh badges) Donor variations increasing the number of cell types increases variability (and lowers reproducibility) Only performed static	Activation of dendric cells by sensitizing chemicals (Lactic acid, Eugenol, Coumarin, Cumene hydroperoxide) in commercially manufactured and self-assembled (primary keratinocytes + THP − 1) RHE model for 24 h in static environment (Schellenberger et al. 2019) Measured lymphocyte surface markers and cytokines for skin sensitising chemicals (2,4-dini-trochlorobenzene, p-phenylenediamine, 2-mercaptobenzothiazole, coumarin, and resorcinol) in primary RHE with peripheral blood mononuclear cells for 9 days in static environment (Frombach et al. 2018)
	Immortalized, i.e., HaCaT, hTERT immortalized primary cells and coculture	Survival and immune competency for up to 17 days in microfluidic environment Model for permeation, corrosion and irritation, sensitization and inflammation	Immortalized cell lines are suspected to behave different than normal skin regarding immune responses Increasing the number of cell types increases variability (and lowers reproducibility)	Measured the effect of chemicals (Nickel Sulfate, Cobalt sulfate, Glycerol and DNCB) and UV stimulation of HaCaT–leukemic monocyte (U937) RHE on the skin barrier in a microfluid system for 17 days (Ramadan and Ting 2016)
	iPSC		To date, iPSC-based RHE and HSE have not been designed with immune cells	
Vascularized skin model	Primary cells and co-cultures	Connects layers and enables oxygen, nutrient and waste flow Shows in vivo*-*like vasculature, cell–cell interaction and neutrophil migration Enhances tissue viability, barrier properties, metabolic activity and immune capacity	Limited studies also incorporate immune cells Increasing the number of cell types increases variability (thus lowers reproducibility) Low proliferative capacity Highly permeable compared to in vivo	Measured on chip the permeation of caffeine and isosorbide dinitrate in fully primary HSE containing HUVECs (Mori et al. 2017) Measured skin irritation of sodium lauryl sulphate using HSE–HUVEC coculture in microfluidic angiogenesis platform (Jusoh et al. 2019) Demonstrated a stable artificial coexistence between integrated liver-skin tissue containing HDMEC in microphysiological system for 14 days (Maschmeyer et al. 2015)
	Immortalized, i.e., HaCaT, hTERT immortalized primary cells and coculture	Connects layers and enables oxygen, nutrient and waste flow Enhances tissue viability, barrier properties, metabolic activity and immune capacity Mixed cell line coculture showed viability for up to 3 weeks	Limited studies incorporate also immune cells In perfusion, stratum corneum appears more uneven Increasing the number of cell types increases variability (and lowers reproducibility)	Measured inflammation and edema induced with tumor necrosis factor alpha in HSE containing HaCaT and primary fibroblasts with HUVEC for 3 weeks (Wufuer et al. 2016)
	iPSC		To date, iPSC-based RHE and HSE have not been designed with vasculature	
HSE with hair follicle	Primary cells	Contain highly CYP active hair follicle within HSE	construction of hair follicles require some expertise, technique (bioprinting), money and time Although vascularized, a static system was used Requires fresh human donations No standardization	Demonstrated a biomimetic approach for generating a hair follicle in a vascularized HSE from cultured primary human cells (Abaci et al. 2018)

Table includes advantages, possible limitations that are based on the reviewed and referenced literature in the text

Another OoC model for skin with higher complexity than a RHE is a Human Skin Equivalent model (HSE, also: Full Thickness, bilayered reconstructed skin model). It consists of an epidermal and dermal compartment (see Fig. 2; Table 1). Commercially available HSE models, T-Skin™, Phenion^®^ Full-Thickness Skin Model, EpiDermFT and Labskin, are derived from primary human cells and allow the investigation of skin metabolism, permeation and wound healing (van den Broek et al. 2017). Self-assembled models also exist with the use immortalized HaCaT and NTERT and iPSC-derived cells (Itoh et al. 2013; Reijnders et al. 2015). To generate a HSE, fibroblasts are integrated into a collagen I scaffold to create a dermal compartment. After coating with adhering collagen fibres, the keratinocytes are seeded on top to form a multilayer. HSE models are particularly suitable for xenobiotic metabolism studies, as the 3D matrix increases the metabolic capacity of the biotransformation enzymes in keratinocytes (Brinkmann et al. 2013). Additional to metabolism studies, HSE are also used for skin permeation studies of topically applied substances due to the increased barrier function compared to RHE (Alberti et al. 2017; Sriram et al. 2018; Schimek et al. 2018). The barrier properties of HSE models can be further improved by adding a hypodermis (subcutis) to advance the barrier function, as demonstrated by Schmidt et al. (2020). This thicker three-layered skin model reduced the permeation, exhibited suitability as an in vitro test system for irritating substances. Moreover, the model was proposed to exploit dermal deposition as a possible new endpoint for chemicals in the lipid-rich hypodermis as there is a fundamental lack of studies for investigating the impact and effect on the pharmacokinetics (Turner and Balu-Iyer 2018; Schmidt et al. 2020). To study sun-associated adverse effects or vitiligo pathogenesis, HSE models are complemented with melanocytes (to treat pigmentation). Commercially manufactured models, MelanoDerm™, epiCS^®^-M and SkinEthic™ RHPE, make use of primary cells in co-culture with normal melanocytes (Lee et al. 2019a). A completely iPSC-derived 3D model has been created but was limited with unexpected low melanocyte count and viability (Gledhill et al. 2015). All before mentioned pigmented HSE have observed limitations, such as pigmentation flaws (complete absence of pigmentation or development of progressive pigmented spots), hypopigmentation or scattered pigmented spots which makes them limitedly recommendable for phototoxicity studies with UV-light exposure on-chip (Germain et al. 2018).

The addition of immune cells to reconstructed skin models allows the study of multicellular immune mechanism and reactions after cutaneous exposure that can potentially initiate allergic contact dermatitis (see Fig. 2; Table 1) (Thélu et al. 2020). The incorporation of the immortalized human acute myeloid leukaemia cell line, MUTZ-3, to derive phenotypically similar Langerhans cells (LC) is widely acknowledged to be valuable (Kosten et al. 2015; Bock et al. 2018). Such coculture models can be created in the lab or obtained from any commercial manufacturer. A RHE model can integrate LC progenitors which differentiate into antigen-presenting LC during tissue reconstruction (SkinEthic RHE 2020). To represent dermal DCs in immune-competent models, primary peripheral blood mononuclear cells (PBMC) and leukemic monocyte THP-1 cells are incorporated (Schellenberger et al. 2019). To date, none of the iPSC-derived skin model incorporates immune cells although iPSC can effectively differentiate into multiple functional lymphocyte lineages (Mathes et al. 2014; van den Broek et al. 2017; Thélu et al. 2020). Overall, all immune cell containing skin models still require more quantitatively defined criteria for reproduceable endpoint studies and are highly complex considering the performance of integrated more simplistic models (Thomas et al. 2019; Baltazar et al. 2020). Therefore, published literature on the application of fully immune competent skin-on-chip is scarce.

To physiologically connect skin tissue layers, the integration of endothelial cells (EC) such as primary derived human umbilical vein endothelial cells (HUVECs), human dermal microvascular EC (HDMEC) or iPSC derived EC are essential. The introduction of blood vessels in the dermis on-chip to simulate the microvasculature demonstrated an enhanced tissue viability, barrier properties, metabolic activity and immune capacity (Materne et al. 2015; Mori et al. 2017). A recent study by Kwak et al. (2020) using a primary cell derived vascular skin tissue on chip, mimicked the neutrophil migration after treatment with sodium lauryl sulphate and, therefore, demonstrated the added value of perfused vascularized models for immune studies. Overall, we conclude the vascularisation of skin tissues in OoC not only enhances functional results compared to static skin equivalents but also allows to study diffusion of chemicals and skin permeability on-chip. Introducing a microvasculature on-chip with good vascular permeability properties could lead to a promising tool in the NGRA toolbox and a platform for higher tier testing to replace the use of ex vivo and animal models (Risueño et al. 2021).

## Application of intestine-on-a-chip in next-generation risk assessment of chemicals

The intestinal system accounts for the nutrient absorption and represents the first barrier of defence to keep harmful agents out of the body and prevent pathogens from entering via the diet. The small intestinal epithelium is characterized by the mucosa that contains circular folds and a dense array of villi to increase the available surface for nutrient uptake from the intestinal lumen. The intestinal mucosa can be divided into three layers, the muscularis mucosae (stroma), the highly vascularized lamina propria and a simple columnar ranged epithelium (Dutton et al. 2019). The small intestinal epithelium primarily consists of enterocytes with absorptive microvilli (> 70%), along with scattered mucus—secreting goblet cells (~ 7%); Paneth cells (5%); stem cells, tuft cells and enteroendocrine cells (together 2%) and covered by a firm layer of glycoprotein mucin (Rao and Wang 2010).

Furthermore, the gut epithelium is characterized by the tight junctional complex consisting of tight junctions and adherent junctions that maintain the barrier properties (Balda and Matter 2008; Sharma et al. 2010). Adding to the barrier property of the intestine, secreted mucus also functions as a stable ecological niche for the residing microbiome to exert enteric defence and food fermentation and breakdown, as well as bile acid metabolization (Liévin-Le Moal and Servin 2006). Moreover, the mucosal epithelium, especially through the M-cells, forms a functional unit with the inherent immune system through the lamina propria which samples luminal material to subsequently present antigens to the dendritic cells (Mestecky et al. 2015; Johansson and Hansson 2016). A protected niche is provided by crypts which are short tubular invaginations. The base of the crypts contain the intestinal stem cells neighboured by Paneth cells which release secretory granules in response to harmful bacteria, lipopolysaccharides (LPS) and cholinergic stimulation to induce an immune reaction (Ganz 2000).

Within the framework of NGRA, OoC intestinal models can be employed as part of the general toolbox to assess gut-related in vitro endpoints focussed on the effect of chemicals on the barrier integrity and interaction of chemicals with the residing microbiome and the local immune system. In addition, OoC intestinal systems can be utilized to obtain data at higher tier targeted testing such as metabolism prediction, binding to proteins and DNA and formation of possible reactive oxygen species. Hence, the development of robust gut-on-chip models may allow the dynamic coculture of human intestinal epithelium cells to closely mimic and tightly control the interaction with microbiota, simulate oral absorption in interplay with physiological and biochemical processes and understand toxicity in the gut tissue.

**Fig. 3:**
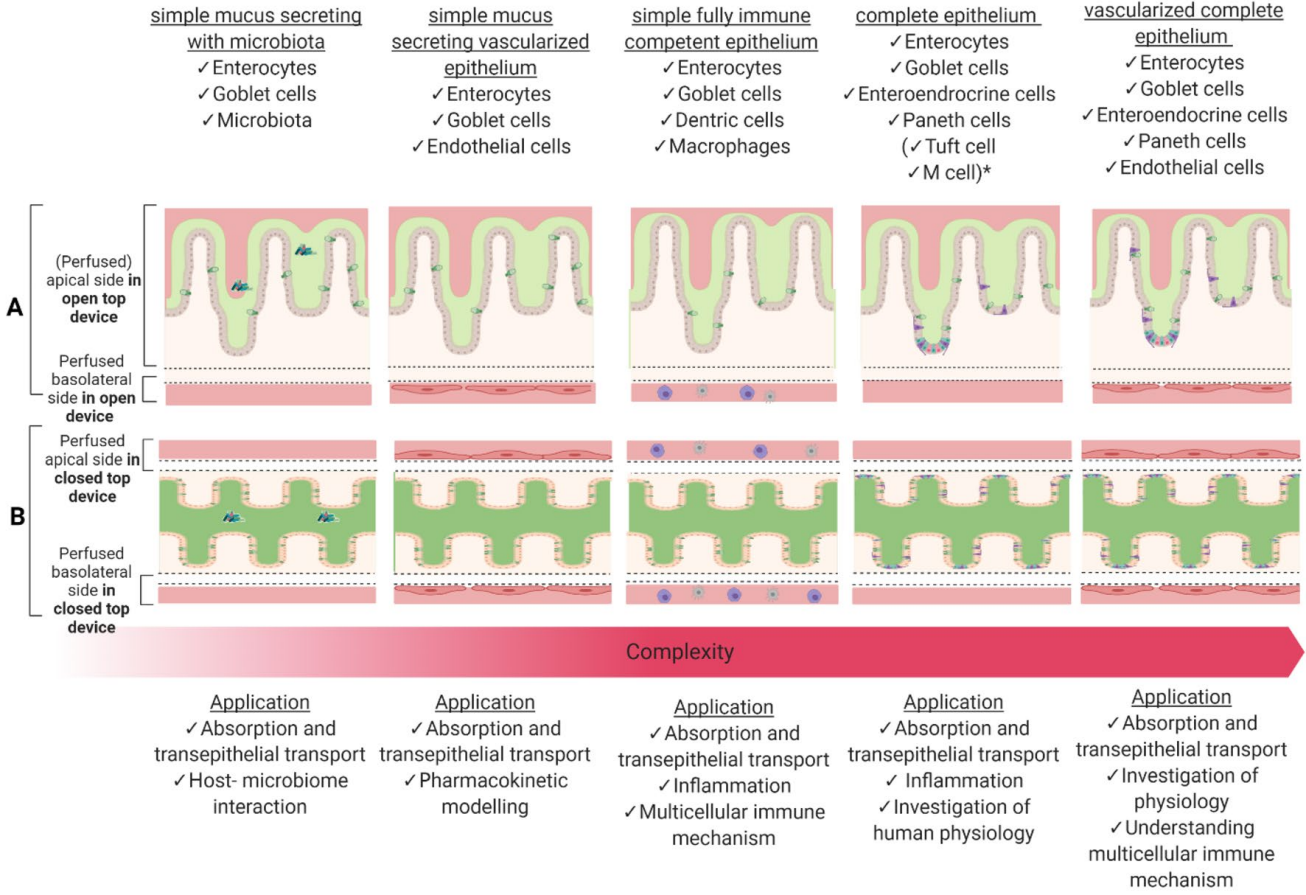
Summary of 3D intestinal tissue models with increasing complexity and their research applicability and predictability for NGRA. The figure depicts two culture designs: **A** only one bottom membrane (top: applicable for cultures using open-accessible layout OoC or two channel closed layout OoC) and **B** three channel closed with perfusion from both sides. (bottom) *only in primary cell cultures

Single intestinal cell type monolayers grown in a static environment have shown to be powerful in vitro models, yet they are limited in the emulation of complex in vivo cell tissue functionality (Costa and Ahluwalia 2019). Therefore, it is important to design human small intestinal tissue models with higher physiological relevance. Here, we will discuss different functional intestinal microtissue models that use membranes, flat or villi-like 3D ECM or other scaffolds in open- and closed accessible OoC devices. Figure 3 and Table 2 describe some of the advanced models that are used for NGRA. These models mostly contain immortalized cells or organoids as it has been technically challenging to culture single primary human intestinal epithelial cells separated from supportive cells (Madden et al. 2018). Therefore, immortalized cell lines and intestinal 3D organoid cultures derived from either intestinal crypts containing endogenous intestinal cells or iPSCs are predominantly used in studies. However, it must be noted that organoids are limited in their lack of supporting cell and tissue types (e.g., endothelial and immune cells) and their closed lumen when cultured within surrounding ECM (Bein et al. 2018).

**Table 2:** Selection of intestinal models that are culturable in microfluidic devices

Type of intestinal cell model	Cell line used	Advantage	Potential limitation	Example application from the literature
Simple (full-thickness) enterocyte epithelium (with mucus secretion)	Immortalized enterocytes, i.e., Caco-2 in coculture	Robust, well-developed microvilli, increased cytoskeleton expression and tight junctional complexes Well-studied and characterized Easy culturable and reproducible Model for absorption and transepithelial transport	Low permeability Low levels of CYP450, especially low CYP3A Increased/decreased gene expression due to genetic mutation Single cell representation	Compared transport of high permeability compounds antipyrine, ketoprofen, and digoxin in dynamic cultured Caco-2 to static transwell model (Kulthong et al. 2020) Studied oral bioavailability of Verapamil and Ergotamin(in)e in flow-through transwell system with Caco-2 and HT-29 MTX (Santbergen et al. 2020) Measured barrier property and mucus secretion in a 3D disk-shaped µ-tissue with Caco-2 cells seeded on top of primary myofibroblasts for 5 days in microfluid system (De Gregorio et al. 2020a)
	Primary cell derived organoid		Separation of the epithelium from the supportive cells may impair function and viability	To date, primary cell-based simple model has not been designed
	iPSC-derived (monoculture)	Demonstrates some toxicokinetic function	Complex, costly and time-intense to induce, differentiate and mature Single cell representation So far low CYP enzyme expression (fetal phenotype)	Generated enterocyte-like cells and measured barrier properties and CYP3A4 induction with 1, 25-dihydroxyvitamin D3 and rifampicin in static environment (Ozawa et al. 2015; Kondo et al. 2020)
Epithelium (with mucus-secretion) and added aerobic microbiome	Immortalized cell line(s), i.e., Caco-2 and co-culture	Close discrepancy in gut physiology Impact on barrier functionality Innovative model to evaluate (anaerobe) host–microbe interaction	Reduction of biological Complexity necessary Potential microbial overgrowth Variability likely due to the complexity Limited studies for anaerobic culturing	Revealed that Shigella infection leverages the intestinal microarchitecture and mechanical forces in Caco-2 tissue on chip (Grassart et al. 2019)
	Primary cells derived organoids	thick mucus layer was produced Bilayered microstructure similar to human colon	separation of the epithelium from the supportive cells may impair function and viability	Studied colon mucus layer accumulation and physiology on chip (Sontheimer-Phelps et al. 2020)
	iPSC-derived (monoculture)	close discrepancy in gut physiology Impact on barrier functionality Model to evaluate host–microbe interaction	Variability likely due to the complexity Potential microbial overgrowth Fragmentation necessary to access lumen Lack of mechanical stimulation	Modelled host–pathogen interaction with *E. coli* using a fragmented stem cell enteroid in monolayer on a chip (Sunuwar et al. 2019)
(Full thickness) Complete epithelium and immune competent epithelium without microbiome	Immortalized cell line(s), i.e., Caco-2 and cocultures	Maintained viable for up to 14 days In vivo comparable transporter and CYP450 activity In vivo comparable permeability Model for absorption, transepithelial transport, inflammation and physiology investigation	Lack other immune competent cells, vascular system, cell lining Variability due to complexity	Measured barrier function using propranolol, mannitol and caffeine; Measured CYP1A1 and 3A4 expression using 3-methyl-cholanthrene and rifampicin in hTERT immortalized primary cells on a primary myofibroblast layer in microfluidic coupled integrated device for 14 days (Chen et al. 2018) Recapitulated and investigated tissue inflammation through neutrophilic infiltration in Caco-2–THP-1 dynamic coculture treated with a combination of lipopolysaccharide and *N*-formylmethionine–leucyl-phenylalanine to mimic presence of bacteria (Gjorevski et al. 2020) Measured barrier permeability and inflammation after exposure to TNFα IL-β, TPCA-1 in Caco-2–HT29–MTX, THP-1 and MUTZ-3 coculture in microfluid condition for 8 days (Gijzen et al. 2020)
	Primary cells derived enteroids or colonoids in coculture	To date, primary cell-based complete epithelium and immune competent epithelium without microbiome have not been designed		
	iPSC/aSC-derived, i.e., organoid	Leak-tight tubules with expression of intestinal markers (susceptible) group specific in vitro models CYP3A4 and MDR1 drug transporter expression higher than in Caco-2 Model for absorption, transepithelial transport, inflammation and physiology investigation	CYP3A4 and MDR1 drug transporter expression lower than in vivo Polarized cells Higher permeability than in vivo Immune competent cells need to be added (not inherent) Variability due to complexity Cost and time intense protocols	Directly differentiated iPSC into intestinal tubules, measured barrier properties and triggered pro-inflammatory cytokines with TNFα, IL-1β and INF-γ for 14 days (Naumovska et al. 2020) Measured after exposure to pro-inflammatory cytokines TNFα and INF-γ the cytotoxicity and permeability in iPSC-derived organoid (Workman et al. 2018)
Vascularized (complete) epithelium	Immortalized cell line(s), i.e., Caco-2 and coculture	Connects layers and enables oxygen, nutrient and waste flow (enhances viability and functions) Model for absorption, transepithelial transport, (complex) inflammation with neutrophil migration and physiology investigation	Low permeability in Caco-2 with HUVEC set up Variability due to complexity	Quantified PK parameters for orally administered nicotine in Caco-2 and HUVEC tissue for 8 days (Herland et al. 2020) Modelled radiation injury-induced cell death and countermeasure drug responses in a Caco-2–HUVEC model (Jalili-Firoozinezhad et al. 2018)
	Primary cells derived enteroids or colonoids in coculture	Connects layers and enables oxygen, nutrient and waste flow (enhances viability and functions) Model for absorption, transepithelial transport, (complex) inflammation with neutrophil migration and physiology investigation In vivo permeability In vivo comparable CYP450 activity	Variability due to complexity of complete epithelium Fragmentation necessary to access lumen Requires the use of time-consuming and labour-intensive procedures Immune cells need to be added (not inherent)	Measured barrier function, drug transporter and CYP3A4 expression and activity after exposure to rifampicin and 1, 25-dihydroxyvitamin D3 in vascularized complete epithelium in microfluid Duodenum-Intestine Chip for 10 days (Kasendra et al. 2018, 2020) Measured barrier function on integrated microfluidic coupled in vascularized full thickness complete epithelium for 14 days (Maschmeyer et al. 2015)
	iPSC/aSC-derived, i.e., organoid	Connects layers and enables oxygen, nutrient and waste flow (enhances viability and functions) Potential model for absorption, transepithelial transport, (complex) inflammation with neutrophil migration and physiology investigation	To date, iPSC-based vascularized (complete) epithelium have not been designed	

Table includes advantages, possible limitations that are based on the reviewed and referenced literature in the text

*aSC* adult stem cell, *TNFα* Tumor Necrosis Factorα, *IL-1β* Interleukin-1β, *INF-γ* Interferon-γ, *TPCA1* IkappaB kinase inhibitor

The simplest in vitro model consists of a columnar enterocyte epithelium which may be cultured with the support of a biological 3D scaffold at the air liquid interface or submerged in the medium. Such a simple model is suitable for intestinal absorption and transport studies as demonstrated in multiple published studies using Caco-2 cells (see Table 2). Caco-2 are considered the gold-standard for investigation of intestinal absorption and transport because of their robustness, well-developed microvilli, increased cytoskeleton expression and tight junctional complexes compared to primary cells. This results in a barrier model with a low permeability for chemicals (Artursson and Borchardt 1997; Hilgendorf et al. 2000). In contrast, biopsy-derived primary human intestinal cells that were separated from supportive muscle cells may show impaired function and viability and are, therefore, not suitable for use in in-vitro intestine models. Stem cell derived models have been limitedly exploited for chemical absorption and transport studies (Madden et al. 2018). Transport studies comparing transwells and microfluidic-perfused cultured Caco-2 tissues, have been performed to study 17 lipophilic dioxin congeners and to compare the transport of the highly permeable compounds, such as antipyrine, ketoprofen and digoxin. According to Kulthong et al., the obtained transport values of the highly permeable chemicals were in line with the compound Biopharmaceuticals Classification System, demonstrating the value of dynamically cultured Caco-2 tissues (Kulthong et al. 2018, 2020). The simple columnar-like epithelium can be expanded by coculturing Caco-2 cells with mucus secreting HT29-MTX goblet cells. A study by Santbergen et al. (2020) successfully coupled a dynamic cultured Caco-2/HT29-MTX model to a chip-based liquid chromatography mass spectrometry for investigation of oral bioavailability of ergotamine. In a different study, in attempt to mimic the lamina propria, De Gregorio et al. (2020a, b) integrated first primary myofibroblasts into their intestinal model with caco-2/HT29-MTX cells cultured on an air–liquid interface. The in vitro model demonstrated an in vivo-like transepithelial resistance but has not been tested for chemical exposure (De Gregorio et al. 2020a, b). Notably, Caco-2 cells contain tighter tight junctions compared to in vivo observations and low levels of cytochrome P450 isoforms, especially CYP3A which is responsible for more than 50% of xenobiotic metabolism in the gut (Kohl 2008). Therefore, an improved Caco-2-based OoC model is needed to better emulate the human intestinal functionality. For instance the addition of mucus secreting HT29–MTX goblet can reduce the permeability and impact cytokine secretion, diffusion of hydrophilic compounds and facilitates adhesion modulation of added microbiome and bacterial components (Hilgendorf et al. 2000; Martínez-Maqueda et al. 2015). The addition of microbiome can serve as an integrative approach to demonstrate host–microbiome interaction in health and disease, such as through inflammation-inducing cytokines and endotoxins but also because of interactions in drug pharmacokinetics and nutrition metabolism (Kim et al. 2016; Jalili-Firoozinezhad et al. 2019; Xiang et al. 2020). However, as reviewed by Elzinga et al. (2019), potential limitations of this complex integrated system include low reproducibility of the (anaerobic) bacterial cultures, potential bacterial overgrowth and a hampered formation of main epithelial cells types and crypts in organoids (Kim et al. 2016; Shin et al. 2020).

A complete epithelium model based either on primary cells or derived from stem cells (see Fig. 3) could provide a holistic model to investigate chemical absorption, metabolism and might provide a tool to study the effect of chemicals. Cui et al. (2020) evaluated the commercial EpiIntestinal™ bioprinted primary microtissue successfully as an ADME tool. In a non-commercial and bioprinted model, Maschmeyer et al. (2015) used an ileum section biopsy to recreate a 3D full-thickness complete epithelium microtissue for an integrated system. In the microfluidic culture, the microtissue was viable and functional for 28 days and expressed physiologically relevant permeability and demonstrated in vivo-like drug transporter and CYP3A4 activity (Maschmeyer et al. 2015). Notably, OoC technology improves the differentiation of iPSC-derived cells into complete intestinal epithelium organoids on-chip, as demonstrated by Naumovska et al. (2020) and Beaurivage et al. (2020). Both studies presented a 3D model with an in vivo-like permeability and a higher drug transporter and CYP3A4 expression as compared to Caco-2 cells. Yet, one major limitation of primary cell and iPSC-derived tissues and organoids is the lack of standardized protocols for metrics and specification to recreate a reliable and predictable performance on-chip (Dutton et al. 2019). Another major limitation of organoids is the overall closed conformation, leading to a restricted apical–luminal access but also the lack of inherent immune competence. Several reports have been published about organoid cocultures with immune cell populations but it is important to acknowledge the challenge of adding additional components to an already complex system (Kim et al. 2020). An alternative is coculturing Caco-2 cells with HT29–MTX, THP-1 and MUTZ-3 as created by Gijzen et al. (2020) who measured barrier permeability and inflammation after exposure to TNFα IL-β, TPCA-1 in dynamic condition to study inflammatory response.

The intestinal models mentioned above can also be expanded by adding vascular cell into the lower membrane surface to introduce a biological barrier for transport studies, physiologically connect layers and to support tissue viability and functionality (Torras et al. 2018). A simple vascularized columnar-like enterocyte epithelium was recreated by Herland et al. (2020) using Caco-2 and HUVEC (see Fig. 3) on a fluidically-coupled organ chip. The endothelium-lined vascular channels in the model allowed for nutrient transport, waste removal and human pharmacokinetic modelling of caffeine. An advanced vitro tissue model by Seiler et al. (2020) used patient-derived human small intestinal cells on a myofibroblast layer with a recreated capillary network. The created tissue was characterized as a translatable ex vivo culture system and demonstrated angiogenic properties after exposure.

Although the studies show great advances in the intestine-on-a-chip field over the past decade, several challenges remain, such as the reproduction of all intestinal layers, especially with stem cell-derived cells. Moreover, intestine-on-a-chip lack a stable integration of a microbiome, and the provision of an intestine-specific environment (e.g., peristaltic, anaerobic etc.). These and more challenges must be overcome to achieve more physiologically relevant and standardized in vitro intestine models (Lee et al. 2019b).

## Application of liver-on-a-chip in next-generation risk assessment of chemicals

The liver is a multifunction organ that coalesces all blood vessels coming from the intestinal tract into the portal vein. The portal vein branches in sinusoids that are comprised of highly permeable sinusoidal endothelial cells (LSECs) surrounded by hepatocytes which are also the main parenchymal cell in the liver (Davenport 2017). A small gap, known as the space of Disse, separates the LSECs from the hepatocytes. The sinusoids are inhabited by non-parenchymal cells such as the hepatic stellate cells (HSC) that help to maintain the ECM) and Kupffer cells which are the liver tissue specific macrophages. So-called bile canaliculi form small channels between the adjacent hepatocytes which secrete the bile. The secreted bile is collected in the bile ducts and transported to the intestine or gall bladder (Lu and Kacew 2002; Ishida 2020).

The liver is crucial organ to be included as an in vitro model as it is the major site for biomodifications of xenobiotics, i.e., Phase I (i.e., Cytochrome (CYP450)) and II (conjugation) metabolism) (Ishibashi et al. 2009). Within the general NGRA toolbox liver-on-chip adds value for the identification of liver specific endpoints, as well as to obtain data at higher tier testing in the ab initio approach application (Berggren et al. 2017). Xenobiotics that enter the liver can undergo biotransformation in which they may become toxicologically active, inactive, or reactive with endogenous macromolecules, potentially resulting in toxicity. Common mechanisms of hepatoxicity include the damage of macromolecules, mitochondrial dysfunction and oxidative stress, the activation of cell death-signalling pathways, modification of cell structure or function, and inflammation. All potentially xenobiotic-induced disturbances may contribute to several pathological conditions, such as steatosis, cholestasis, fibrosis and cirrhosis (McGill et al. 2015). Particular for the liver, the in vitro assessment of the end-points is complicated as the in vivo sinusoidal cell environment and functions are not homogenous along the portal–central vein axis. Depending on spatial location (zone) along this axis, an oxygen and metabolic enzyme gradient is created. Consequently, the present functional gradient in substrate metabolism, synthesis, storage and excretion affects the xenobiotic metabolism, resulting in a site-specific hepatic toxicity, altering gene expression and cell functions (Lu and Kacew 2002; McGinnity and Grime 2017; Soto-Gutierrez et al. 2017; Ahn et al. 2019; Ishida 2020).

**Fig. 4:**
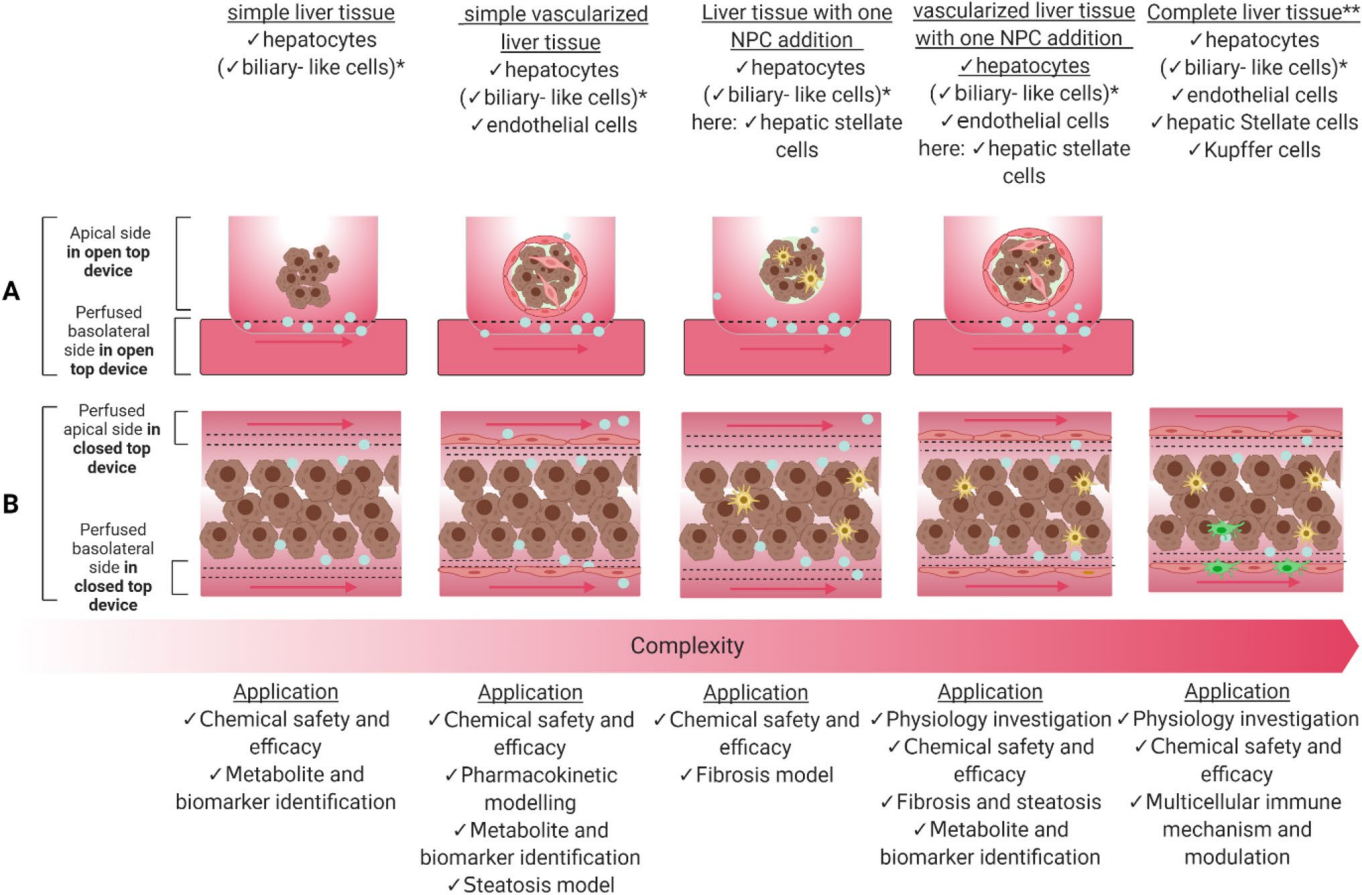
Summary of 3D liver tissue models with increasing complexity and their application in NGRA. The figure depicts two tissue approaches, **A** only a bottom membrane (top; applicable for cultures using open-accessible devices) and **B** three channel closed-accessible designs with perfusion from both sides. (bottom) *only in HepaRG, primary cell cultures and iPSC **does not exist (yet) as spheroid

Predictive liver in vitro models are highly demanded to evaluate biotransformation and mechanism-based hepatoxicity. Dynamic culturing holds a great promise to expand the predictive capacity by facilitating the emulation of liver-specific functions and spatial gradient variation and to perform targeted testing and biokinetic refinements (Berggren et al. 2017; Kang et al. 2018; Ehrlich et al. 2019; Ahn et al. 2019). Figure 4 and Table 3 depict liver models with increasing biological complexity, cultured in different OoC layouts and containing differently originated cells. Notably, three liver tissue formats are frequently used in OoC: hydrogel scaffolded 3D tissues, pre-cultured spheroids and 2D monolayers.

**Table 3:** Selection of liver models that are culturable in microfluidic devices

Type of liver tissue	Cell line used	Advantage	Potential limitation	Example application from the literature
Conventional 2D culture (e.g., Sandwich, Monolayer)	Immortalized, i.e., HepG2, HepaRG	No specialized system/equipment needed to create monoculture layer Elaborate protocols and availability Good experimental reproducibility Easy to culture and low in cost	Lack of supportive NPCs in monocultures Lack of physiological relevant 3D architecture Lack of polarity Typically lower drug metabolism enzyme activity Lack of physiological relevant 3D architecture	Compared gene expression and biotransformation activity after TCDD and rifampicin exposure in dynamically a 2D HepaRG sandwich culture to static culture (Duivenvoorde et al. 2021)
	Primary cell line	No specialized system/equipment needed to create monoculture layer Elaborate protocols and availability of cryopreserved cells Comparable to in vivo CYP phase II enzyme activity in sandwich and coculture	Lack of supportive NPCs in monocultures Lack of polarity Lack of physiological relevant 3D architecture	Measured mRNA levels and xenobiotic metabolism after treatment with Phenacetin and Midazolam in long-term hepatocyte monolayer on microfluidic biochip for 13 days (Jellali et al. 2016) Measured the CYP expression after TCDD and rifampicin exposure in high-throughput OoC platform using PHH in 2D culture (Azizgolshani et al. 2021)
	iPSC-derived monoculture	Suitable for genetic disease investigation Rapid progress in differentiation and maturation protocols	Lack of supportive NPCs in monocultures Induction, differentiation and maturation requires expertise, money and time Limited expression of some liver-specific genes, fetal phenotype	Measured long-term toxicity with Amiodarone, Troglitazone, Aflatoxin B1, Ximelagatran in human pluripotent stem cell derived hepatocyte-like cells in static environment (Holmgren et al. 2014)
3D Simple spheroid/organoid (with NPC addition)	Immortalized, i.e., HepG2, HepaRG and cocultures	3D and flow improves microenvironment and function Elaborate protocols and techniques good experimental reproducibility Easy to culture and low in cost HepaRG contain some liver specific functions comparable to PHH Potential model for chemical safety, efficacy, metabolite and hepatoxic biomarker identification	necrotic core likely in non-size controlled self-assembled spheroid aggregates No validated standard on how to produce Cocultures with NPCs Function of hepatocytes is highly dependent on NPC support choice and the random distribution can express in morphologic and functional instability Poor expression of some adult liver functions (e.g., CYP Phase II enzymes, transporters, p53)	Measured DILI and Interleukin 6 secretion in PHH–KC spheroid after treatment with lipopolysaccharide and trovafloxacin in static condition (Li et al. 2020) Functionally coupled HepaRG–HSC spheroid with pancreatic islets on a chip to model diabetes type 2 after repeated exposure to high glucose levels (Bauer et al. 2017) Studied anticancer drug (5-fluorouracil) cytotoxicity based on long-term HepG2 spheroid culture in microfluidic system (Zuchowska et al. 2017) (spheroids formed in device)
	Primary cell line and cocultures	Elaborate protocols and availability of cryopreserved cells Comparable to in vivo transporter and CYP phase II enzyme activity Potential model for chemical safety, efficacy, metabolite and hepatoxic biomarker identification	necrotic core likely in non-size controlled self-assembled spheroid aggregates No standard on how to produce cocultures with NPCs Function of hepatocytes is highly dependent on NPC support choice and the random distribution can express in morphologic and functional instability	Measured in multiple PHH models with non-parenchymal cell addition metabolism, bioactivation and cell-interactions in static environments. Repeated exposure to control set of CYP inducers and Acetaminophen (Bell et al. 2016, 2018, 2020)
	aSC/iPSC-derived (Organoid)	Fully differentiated and matured: comparable to in vivo hepatocytes Rapid progress in differentiation and maturation protocols potential model for chemical safety, efficacy, metabolite and hepatoxic biomarker identification	function of hepatocytes is highly dependent on NPC support choice and the random distribution can express in morphologic and functional instability No standard on how to produce cocultures with NPCs Differentiation and maturation require expertise and time Still limited expression of some liver-specific genes (fetal phenotype) All hepatic iPSC-cell types need to be induced, differentiated and matured separately	Characterized patient-specific drug screening with iPSC derived hepatocyte-like cells using validated CYP inducers (7-ethoxyresorufin; Coumarin; Testosterone; Bupropion; S-mephenytoin; Dextromethorphan) perfused on-chip for over 28 days (Schepers et al. 2016) Achieved in situ differentiation of hepatic iPSCs on dynamic cultured micropillar chip into hepatocytes and Cholangiocyte and showed dose- and time dependent hepatoxcitiy response to Acetaminophen (Yaqing Wang et al. 2018a, b)
3D Vascularized spheroid/organoid (with NPC addition)	Immortalized, i.e., HepG2, HepaRG and cocultures	Elaborate protocols and human material availability Improved supply of nutrients and oxygen; waste removal Increased performance liver specific functions compared to non-vascularised models Model for steatosis, fibrosis and chemical safety (e.g., biomarker)	No validated standard on how to produce cocultures with NPCs Non-defined architecture can impact viability and functionality	Established NADFL model with free fatty acid supplementation (palmitic and oleic acid) on HepG2–HUVEC tissue in microfluid condition; Reversed condition with antisteatotic drugs (Metformin and pioglitazone) (Lasli et al. 2019)
	Primary cell line	elaborate protocols, techniques and human material availability Improved supply of nutrients and oxygen; waste removal Increased performance liver specific functions compared to non-vascularised models Model for steatosis, fibrosis and chemical safety (e.g., biomarker)	Non-defined architecture can impact viability and functionality No validated standard on how to produce cocultures with NPCs	Modelled DILI dose response to Trovafloxacinin in bioprinted spheroid containing PHH–HSC–HUVEC (Nguyen et al. 2016)
	aSC/iPSC-derived (Organoid)	Improved supply of nutrients and oxygen; waste removal Potentially increased performance liver specific functions after full maturation compared to non-vascularised models Potential model for steatosis, fibrosis and chemical safety (e.g., biomarker)	Non-defined architecture can impact viability and functionality No standard on how to produce cocultures with NPCs Differentiation and maturation require expertise and time iPSC-cell types need to be induced, differentiated and matured separately	Demonstrated improved maturity of human pluripotent stem cell-derived hepatocytes without exposure in 3D culture with LSEC–HSC and Cholangiocyte primary cells in static condition (Ardalani et al. 2019)
3D simple scaffolded tissue (with NPC addition)	Immortalized, i.e., HepG2, HepaRG	Good experimental reproducibility Easy to culture and low in cost HepaRG contain some liver specific functions comparable to PHH Potential model for chemical safety, efficacy, metabolite and hepatoxic biomarker identification	Function of hepatocytes is highly dependent on NPC support choice and the random distribution can express in morphologic and functional instability No standard for NPC cocultures Poor expression of some adult liver functions (e.g., CYP Phase II enzymes, transporters)	Compared single-organ exposure to fluidic coupled (gut) exposure with CYP1A1 (3-methyl-cholanthrene) and CYP3A4 (rifampicin) inducers in HepG2 tissue for 14 days in microfluid condition (Chen et al. 2018)
	Primary cell line	Longer maintainable and viable than 2D monolayers and static cultures Model for chemical safety, efficacy, metabolite and hepatoxic biomarker identification	No validated standard on how to produce cocultures with NPCs Function of hepatocytes is highly dependent on NPC support choice and the random distribution can express in morphologic and functional instability	Created a non-alcoholic steatohepatitis model with PHH–HSC–KC culture for 2 week on-chip for drug discovery, biology exploration and compound screening (Kostrzewski et al. 2020) Established NADFL model with free fatty acid supplementation (palmitic and oleic acid) and then measured activity of CYP1A2 (Tacrine), CYP2C9 (Diclofenac), CYP2D6 (Bufuralol) and CYP3A4 (Midozalam) in PHH tissue under perfusion; Reversed condition with antisteatotic drugs (Metformin and pioglitazone) (Kostrzewski et al. 2017) Study the regulation of cytochrome P450 3A4 isoform (CYP3A4) activity by chronic interleukin 6 (IL-6)-mediated inflammation in PHH–KC coculture over 2 weeks and impact of Tocilizumab treatment on CYPs (Long et al. 2016)
	aSC/iPSC-derived (monoculture)	Shear stress improves overall performance and fetal–phenotype Fully differentiated and matured stem cells: comparable to in vivo hepatocytes Symbiotic progress in induction, differentiation and maturation protocols	Function of hepatocytes is highly dependent on NPC support choice and the random distribution can express in morphologic and functional instability Differentiation and maturation require expertise and time Potentially limited expression of some liver-specific genes (fetal phenotype) iPSC-cell types need to be induced, differentiated and matured separately	Differentiated human iPSc in perfused bioreactor and measured activities of CYP3A4, CYP1A2, CYP3A7 through midazolam, phenacetin and bupropion exposure after 18 days in culture (Meier et al. 2017)
3D vascularized scaffolded tissue (with NPC addition)	Immortalized, i.e., HepG2, HepaRG	Elaborate protocols and human material availability Improved supply of nutrients and oxygen; waste removal Increased performance liver specific functions compared to non-vascularised models	no validated standard on how to produce cocultures with NPCs Non-defined architecture can impact viability and functionality-no validated standard on how to produce cocultures with NPCs Non-defined architecture can impact viability, morphology and functionality	Investigated hepatoprotectant effects of tiopronin, bifendatatum, and glycyrrhizinate after acetaminophen exposure in HepG2–HUVEC–HSC–KC coculture (Deng et al. 2020) Compared dymanic and static cultured HepaRG–HUVEC–HSC–PMBC tissue and measured CYP3A4 expression using Midozalam in perfused device for 4 days (Rennert et al. 2015)
	Primary cell line	Improved supply of nutrients and oxygen; waste removal Increased performance liver specific functions compared to non-vascularised models	No validated standard on how to produce cocultures with NPCs Non-defined architecture can impact viability, functionality and morphology	Studied non-alcoholic steatohepatitis pathogenesis using palmitic with oleic acid and lipopolysaccharide, pharmacologic intervention with elafibranor in PHH–LSEC–HSC–KC coculture (Freag et al. 2021) Recapitulated immune response to TGF-β in continuously zonated vascularized tissue containing PHH–LSEC–HSC–KC (Li et al. 2018)
	aSC/iPSC	Increased performance liver specific functions compared to non-vascularised models Shear stress improves overall performance and fetal-phenotype Fully differentiated and matured stem cells: comparable to in vivo hepatocytes Symbiotic progress in induction, differentiation and maturation protocols	No standard on how to produce cocultures with NPCs Function of hepatocytes is highly dependent on NPC support choice and the random distribution can express in morphologic and functional instability iPSC-cell types need to be induced, differentiated and matured separately (solution: cell line coculture)	Analysis of reproducibility and robustness of a human microfluidic four-cell liver acinus microphysiology system (LAMPS) after exposure to Terfenadine, Caffeine, lipopolysaccharide, Rosiglitazone, Pioglitazone, Troglitazone, Tolcapone, Trovafloxacin and Mifepristone using a iPSC-derived hepatocytes–HUVEC–HSC–KC coculture (Sakolish et al. 2021) Created an immune competent high-throughput liver model with iPSC-derived hepatocytes-HMEC1 and THP1 cell tissue and measured CYP capacity using phenacetin (CYP1A1/2), Coumarin (CYP2A6), Diclofenac (CYP2C9),Terfenadine (CYP3A4), phenolphthalein (glucoronidation), and hepatoxins troglitazone and Aflatoxin B1 (Bircsak et al. 2021)

Table includes advantages, possible limitations that are based on the reviewed and referenced literature in the text

*aSC* adult stem cells, *NPC* non-parenchymal cells, *PHH* primary human hepatocytes, *HSC* hepatic stellate cells, *KC* Kupffer Cells, *HUVEC* human umbilical vein endothelial cells, *LSEC* Liver sinusoidal endothelial cells, *TGF-β* tumor growth factor β, *NAFLD* non-alcoholic fatty liver disease, *TCDD* 2,3,7,8-Tetrachlorodibenzo-p-dioxin

Single cell type monolayers cultured on a coated microporous membrane are still a frequently used tissue format in OoC. Sandwich- and micropatterned (co)cultures are both metabolically competent and have proper localisation of basolateral and canalicular transporters with functional bile networks (Swift et al. 2010; Beckwitt et al. 2018). Recently, Duivenvoorde et al. (2021) cultured dynamically a 2D HepaRG sandwich culture and demonstrated successfully improved gene expression and biotransformation activity compared to a static culture. Azizgolshani et al. (2021) came to a similar result when measuring the CYP expression in real time with their high-throughput OoC platform using primary hepatocytes (PHH) in 2D culture. Notably, sandwich cultures are restricted by their flat histology for physiological-relevant coculture with non-parenchymal cells (NPC), whereas micropatterns may constrain the cell morphology (i.e., shape) and come partially with extra manufacturing costs and material concerns (D’Arcangelo and McGuigan 2015; Zhang et al. 2017b).

The recent OoC culture advances enables 3D spheroids models to remain viable for much longer period of time than conventional sandwich cultures, allowing for repeated exposure studies (Ramaiahgari and Ferguson 2019). Spheroids can be easily seeded and cultured in open-accessible OoC device (see Fig. 4 top) and generated with several techniques prior seeding, such as hanging drop, spinner flasks, cell culture on ultra-low attachment surfaces and scaffold-based micromolding (Ma et al. 2018). Larger aggregates exhibit limitations in mass transport for nutrients and oxygen diffusion easily causing a necrotic cell death inside the spheroid core due to impaired cell division, as well as heterogenous viability and function. A study in static condition by Bell et al. (2016) stated a high viability and functionality of self-assembling primary human hepatocytes (PHH) spheroidal aggregates but also difficulty to maintain a uniform size and cluster of cells in non-adhesive plates (Ma et al. 2018; Underhill and Khetani 2018). Plate-based and hydrogel micromolding and bioprinting address the limitation of spheroid size variability by directing the assembly. The iFlowPlate™ by Lin et al. (2021) currently offers an approach to produce scalable perfusable vascularized liver spheroids for OoC without bioprinting.

A simple iPSC-derived hepatocyte-like (HCL) organoid for an in-lab assembled OoC was established by Schepers et al. (2016) which exhibited genotypic CYP450 activity which could be maintained for 28 days. A big leap towards a more complex liver model was taken by Leite et al. (2016) and Maschmeyer et al. (2015) through the addition of primary human hepatic stellate cells to a primary human hepatocyte (PHH) culture in the TissUse OoC device. Maschmeyer tested the hepatic biotransformation by repeated troglitazone treatment, whereas Leite investigated chemical-induced HSC activation and fibrosis using Allyl alcohol and Methotrextate (Maschmeyer et al. 2015; Leite et al. 2016). In a different study, using a PHH-Kupffer cells coculture in static condition, Li et al. (2020) investigated the role of Kupffer cells in inflammation and drug-induced liver injury (DILI). The immune competent liver spheroid model demonstrated the importance of Kupffer cells in DILI by evaluating the signalling pathways after treatment with lipopolysaccharide and trovafloxacin. In another spheroid model by Lasli et al (2019), endothelial cells of primary origin were incorporated to form vascularized liver spheroids. These authors precultured in pyramid-shaped microwells HepG2 with HUVECs to size-select the self-assembled spheroids. After collection and culturing in microfluid condition, the spheroids showed a stable phenotype to model hepatic steatosis induced with palmitic and oleic acid. Another biologically complex model was developed by Ardalani et al. (2019), using iPSC-derived hepatocytes and endothelial cells with hepatic stellate cells and Cholangiocyte primary cells in static condition. The developed vascularized spheroid model showed that coculture with endothelial cells improve hepatic functionality, but the model still expressed fetal markers and immature functions compared to primary cells. Eventually, the authors conclude that the integration of the aggregates in OoC can potentially improve the liver model (Ardalani et al. 2019).

A final culturing format is the culturing of liver cells in a 3D scaffold on chip. For this, liver cells are mixed with scaffolding proteins (ECM) to self-assemble a 3D structure within a closed OoC culture compartment. As to be seen on the bottom of Fig. 4, the cell-ECM culture mixture is separated by protective microporous membrane from the perfusion channels (Jang et al. 2019a, b). A simple liver tissue was created by Jang et al. (2019a, b), who differentiated HepaRG progenitor cells without dimethyl sulfoxide directly on chip. The study demonstrated the major advantage of this liver tissue type, the simplicity to study Phase I and II metabolism, transport and hepatoxicity. However, the limitation of this simplicity is the reduced biology due to the lack of supporting non parenchymal cells that might impact effects after chemical exposure. In the liver, endothelial cells represent the most abundant NPC and form the crucial permeable blood–parenchymal barrier. Vascularized liver tissue models may incorporate primary human endothelial cells such as LSEC, HUVECs or iPSC-EC into the (upper and) lower perfusion channel to recreate an in vivo*-*like permeable barrier. A simple vascularized tissue was established by Herland et al. (2020), containing a PHH–LSEC coculture that was incorporated into a multi-organ-chip. Within this system, this rather simple tissue could successfully mimic the first-pass-metabolism of nicotine and quantitatively predicted human pharmacokinetic parameters for in silico modelling. In pathological conditions, LSECs play a key role in the initiation and progression of chronic liver diseases in interplay with HSC and Kupffer cells (Poisson et al. 2017). HSC mediate the balance of inflammation, the tissue generation after DILI while also facilitating cell–cell communication between hepatocytes and endothelial cells, whereas Kupffer cells play a major role in inflammation and immune responses (Kasuya et al. 2011). Long et al. (2016) studied in a PHH–Kupffer cell coculture the regulation of cytochrome P450 3A4 isoform (CYP3A4) activity by chronic interleukin 6 (IL-6)-mediated inflammation over 2 weeks and the de-suppressed CYP3A4 activity of Tocilizumab exposure in presence of IL-6. The most complex liver tissue model that incorporates all four major liver cell types at in vivo ratios (here: Fig. 4 as complete liver tissue) was established by Vernetti et al. (2016) and called Liver Acinus MicroPhysiology System (LAMP). The focus of that study with the LAMP model was to measure CYP and UGT activity over 28 days in cells of primary source. Later, Li et al. (2018) recapitulated clinically relevant tissue responses for experimental modelling of liver physiology and (immune) diseases, as well as ADME/TOX using the same model. At this complexity the tissue might mimic elaborate (immune) diseases but becomes also more prone to variabilities, as no validated standards exist to engineer and scale these complex NPC cocultures for the characterization of key events in NGRA. However, Sakolish et al. (2021) showed that LAMPS can be a robust and reproducible in vitro liver model in dynamic culture. The improved model performance was in vivo-comparable when the tissue was seeded with either primary human hepatocytes or iPSC-derived hepatocytes.

## Functional integration of tissue systems by fluidic coupling

The next major step in OoC technology is the microfluidic functional coupling of individual organ-compartments to a multi-organ-chip (Sang Hun Lee and Jun 2019; Vernetti et al. 2017). Multi-organ-chip have great potential to improve the NGRA toolbox as the different incorporated organ tissues will affect the pharmacokinetic and pharmacodynamic properties of circulating chemicals. This novel but complex approach will facilitate the simulation of absorption (i.e., skin or intestine), subsequent first-pass metabolism and/or hepatic bioactivation, transport to the target-organ(s) (ADME) (see Table 4). Although the manufactured OoC devices differ in design for their mimicked function (i.e., air–liquid culturing), most platforms allow their tissue to be fluidically linked to enable dynamic tissue–tissue communication through the secreted soluble factors and extracellular vehicles (Ronaldson-Bouchard and Vunjak-Novakovic 2018; Wu et al. 2020). Depending on the culture set up, the tissue locations are fluidically connected with passive flow or active flow via a pump with tubing or a monolithic design (Renggli and Frey 2020; Zhang et al. 2020). An alternative fluid exchange displays transferring fluids with an automated liquid-handling instrument between reservoirs. Multi-organ-chip offer undoubtedly a diverse spectrum of applications in NGRA and are expected to provide novel solutions in the field of New Approach Methodologies (Punt et al. 2020). Technical challenges that still need to find a solution include the development of a device that considers at the same time different organ flow patterns and functions (i.e., peristalsis, elongation, local pressure) whilst using an appropriate material (e.g., non-binding, biocompatible) but also facilitates long-term culture (e.g., decrease risk of contamination and trapped air bubbles) (Renggli and Frey 2020). Besides, current biological constraints range from an appropriate and physiological-relevant tissue scaling and stability, to a common medium composition for circulation (for coculture connection and feeding), as well as the selection and creation of assays to evaluate the culture tissues separately (Bovard and Sandoz 2019; Picollet-D’hahan et al. 2021). However, current multi-organ-chips can already emulate key aspects of an in vivo human environment and mimic organ–organ interaction and ADME processes which was previously only available through in vivo models. Therefore, more advances in device manufacturing, fit-for-purpose and validated assays and protocols is highly expected as multi-organ-chip system developers and users are gaining scientific experience (Marx 2020).

**Table 4:** Selection of studies using microfluidic coupling to co-organ culture with varying applications. Studies made use of different commercially available devices or self-assembled platforms and are focused on skin, gut and liver co-organ cultures

Integrated tissues	Example application	References
Skin–liver	Integrated skin tissue into two-organ chip for permeation study with possibility to extended model for in vitro substance testing including liver	Schimek et al. (2018) Tao et al. (2021)
Intestine–liver	First-pass metabolism of ethanol	De Gregorio et al. ([Bibr CR33][Bibr CR34])
Intestine–liver	Acetaminophen absorption and metabolism	Marin et al. (2019)
Intestine–liver	Quantitative in vitro pharmacokinetic study	Tsamandouras et al. (2017)
Intestine–liver–kidney	Quantitative prediction of human pharmacokinetic and toxicity	Herland et al. (2020)
Intestine–liver–kidney	Establishment of exposure-response relationship for pharmacodynamics and toxicity	Maass et al. (2017)
Intestine–liver–brain–kidney	Autologous induced pluripotent—stem cell derivation from same donor	Ramme et al. (2019)
Skin–liver	Characterization of application scenario-dependent pharmacokinetics and pharmacodynamic properties of permethrin and hyperforin	Kühnl et al. (2021)
Skin–heart–liver	Evaluation of topical drug delivery	Pires De Mello et al. (2020)

## Challenges and prospects for OoC technology and NGRA on-chip with skin, intestine and liver tissues

Organ-on-chip is a rapidly evolving technology that offers versatile systems to mediate the formation of functional tissues and organs for different research applications (see Fig. 1). All reviewed commercially available devices offer significant technical advantages to culture and investigate biologically improved organ tissue models. Yet, there are clear needs and challenges that must still be addressed from the initial hardware development until the final user application as also pointed out by the organ-on-chip-in-development (ORCHID) initiative roadmap (Mastrangeli et al. 2019; Piergiovanni et al. 2021). Aligning with the ORCHID initiative, we conclude that the device specifications must be addressed first. This includes the search for the ideal (hybrid) materials for devices and scaffolds that can enable appropriate cell cultures with low chemical adsorption and absorption and biocompatibility. In addition, novel (hybrid) materials should be flexible to allow for physical strain to be included (i.e., stretchable membranes), while optical transparency should remain present for cell imaging (i.e., microscopy).

While a diversity of commercially available OoC devices exist, there is no ideal versatile hardware layout. Some of the devices have external dimensions that are comparable to routinely used labware which allows easier integration into routine lab practices. We recognize that different research questions, and specific tissues culturing demands (i.e., for skin, intestinal and liver tissues) require different OoC device designs. For the barrier skin open-accessible devices might be desirable for air liquid culturing, while for the intestine OoC models both open and closed configurations might be interesting. Open-accessible tissue compartments offer better access for pipetting, layering, air–liquid interfacing or space for bigger cell aggregates, whereas the flow can be less controlled. In contrast, closed culture compartment can mimic better mechanical forces such as flow and stretch and may allow anaerobic intestinal culture.

An important aspect of OoC devices is the current lack of versatile microfluidic perfusion in the devices. Flow can be actively induced through directly integrated and plug-in pumps or passively through gravity-drive. Passive perfusion enables flow without additional tubing, whereas devices using pumps allow for a more controlled induction of shear stress and facilitate fluidic connection to different organ tissues. Nevertheless, there are still technical challenges to face for the hardware that include a stable fluid connection without bubbles and sterility of tubing. While often addressed as a concern, chemical sorbing to the materials used have only limitedly been studied so far. Data and knowledge on the absorption of chemicals on to the fabrication material needed to ascertain the acceptance of OoC models as NGRA toolbox for the toxicological hazard characterization of chemicals.

Yet, OoC tissue models have already advanced the biology of human in vitro tissue culturing. The combination of both, 3D architecture and fluidic flow, has shown great impact on cellular characteristics such as on the morphology, viability, differentiation, metabolic and enzymatic capacity, as well as transporter and gene expression levels. These improvements strengthen the relevance of OoC technology as the advances have been observed with several cell lines and cell types. The next step towards creating advanced 3D cultures and incorporation of stem cell derived tissues on-chip for a future NGRA are promising but also show the need to standardise advanced organ tissue culturing for human health effect assessment.

For the skin tissue, models demonstrated that OoC offer an improved approach to assess the safety and efficacy of topically applied consumer products to assess endpoints, such as permeation, irritation and corrosion, phototoxicity, as well as skin sensitization and inflammation. For the investigation of these endpoints a range of different skin models were established and assessed ranging from single cell type model such as RHE to HSE with additional cell types and appendices (see Fig. 2). Specifically for skin-on-chip, the new adopted OECD guidelines for endpoint testing (OECD 2021a, b, c) will gain importance to harmonise the assessment for chemical safety information.

For the dynamically cultured intestinal tissues an advanced understanding of the permeability, absorption and transport orally ingested compounds has been shown. The introduced in vitro intestinal tissues ranged in biological complexity, from a simple columnar-like enterocyte epithelium up to a vascularized complete epithelium to target different NGRA applications (see Fig. 3). Notably, dynamic culturing of a mucus secreting epithelium with a microbiome might address the need for a host–microbiome interaction model in health and disease. Specifically, intestine-on-chip will further advance studies focussing on oral delivery, toxicokinetic, nutritional metabolism and disease development as it can simulate better the complex in vitro environment rather than static monolayers.

For a dynamically cultured liver models, all approaches improved the study of molecular mechanism impacting efficacy and safety of test chemicals but showed a successful investigation of improved bioactivation, as well as a better emulation of physiological and pathological mechanisms. The long-term maintenance and function was positively impacted by the coculture with vascular cells in all the three, liver, skin and intestine, models. In combination with shear stress, vascular cells introduced a selective biological barrier that mediates tissue homeostasis by supplying the tissues with nutrients and oxygen. Especially for applying liver-on-chip in NGRA, vascularisation is suited to enable long-term stability of the tissue culture for repeated exposure, as well as to investigate toxicity mechanisms through biomechanical factors, extracellular (or diffusible) signalling molecules and cell–cell interaction (Wang et al. 2018a, b).

Overall, we conclude that dynamic culturing is not only revolutionising in vitro tissue culturing on-chip but also provides a novel solution for the NGRA toolbox to characterise chemicals and their specific modes of action for toxicity, as well as fill and refine data gaps without generating new animal data (Punt et al. 2020; Hatherell et al. 2020). For the NGRA framework, the reviewed and selected studies demonstrate how OoC provides the opportunity for human-centric toxicokinetic- and dynamic studies to fill and refine data gaps, either as a single emulated organ or as complex fluidically linked multi-organ system. The obtained results can be used to in integrative PBK models and to perform quantitative in vitro to in vivo extrapolations for chemical hazard characterization. However, before the framework shift and regulatory acceptance, the novel technology must still address biological questions that come with device design (e.g., choice of material, layout, perfusion) and tissue engineering (e.g., organ scaling, blood substitutes, chronic and systemic toxicity, culture and assay protocols) to qualify as reliable and validated fit-for-purpose-system. In the long run, OoC bears the potential to not only outperform traditional in vitro methods but also to accelerate the transition to human-based predictive chemical safety assessment.
